# Mating Type Determination in *Tetrahymena*: Last Man Standing

**DOI:** 10.1371/journal.pbio.1001522

**Published:** 2013-03-26

**Authors:** Richard Robinson

**Affiliations:** Freelance Science Writer, Sherborn, Massachusetts, United States of America

*Tetrahymena*, a single-celled protist, is a eukaryote, just like you and me. But that's about where the similarity ends. Each *Tetrahymena* cell contains not one, but two nuclei. The diploid germline nucleus remains transcriptionally silent throughout asexual reproduction, while the somatic nucleus is transcriptionally active. Most especially, *Tetrahymena* do sex differently from us—there are not two, but seven mating types (I to VII), and any *Tetrahymena* cell can mate with a cell of any type but its own.

**Figure:**
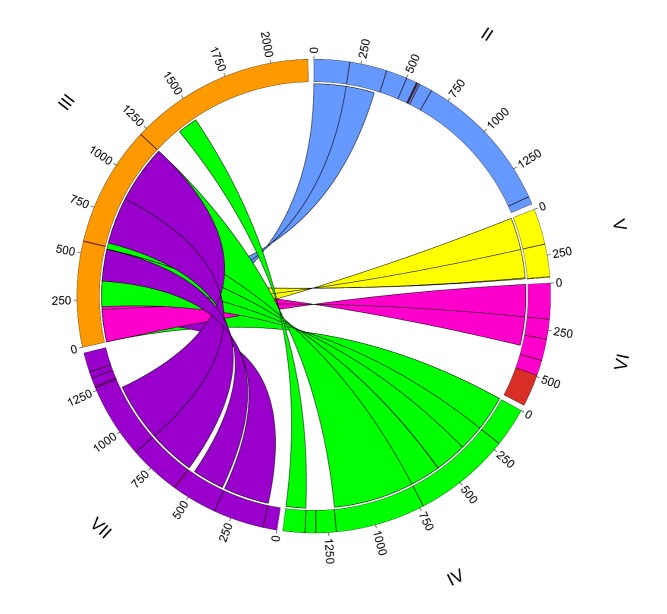
Assembling a mating type gene in *Tetrahymena*. Ribbons show how each incomplete *MTB* gene (color coded) is joined to the end of the only complete gene (III, orange). Image credit: Michael J. Lawson.

The existence of the seven mating types has been known since the 1950s, but the genetic basis of mating type determination has remained a mystery until now. In this issue of *PLOS Biology*, Marcella Cervantes, Wei Miao, Eduardo Orias, and colleagues show that incomplete gene pairs for each type are arranged in a linear array within the germline nucleus. During the mating process, completion of one gene pair by joining non-contiguous DNA segments, and stochastic elimination of all the others within the new somatic nucleus, fixes the mating type of the offspring.

To search for genes that might be involved in mating, the authors starved the cells, a necessary step before conjugation (sex). During conjugation, gamete nuclei fuse and then divide mitotically to form both germline and somatic nuclei; the old somatic nucleus from each parent is then destroyed. The new somatic nucleus undergoes wholesale genomic rearrangements that result in, among other things, determination of mating type.

The authors took RNA sequences from starved mating type V and VI cells and mapped them onto the *Tetrahymena* somatic genome. They found two adjacent genes that were not expressed during growth, and were expressed only in mating type VI (not V) during starvation, and showed that knocking out either one prevented conjugation, suggesting they were involved in mating. They called the genes *MTA* and *MTB*. In the somatic nucleus, *MTA* and *MTB* were arranged head to head, each containing an exon at its distal end that encodes a transmembrane domain. Since two *Tetrahymena* must contact each other to sense a mating type difference, it stood to reason they might employ membrane proteins to distinguish between self and non-self mating types, strengthening the case for the involvement of the two genes.

Next, they searched the germline genome sequence for the type VI *MTA* and *MTB* sequences, and got a surprise. They found a 91-kilobase region of the genome in which the transmembrane portion of each gene had not one, but *six* separate matches, while the remaining portion matched only once. They tried MTA and MTB sequences from the remaining types, and found that the mating type–specific region of each gene matched a different site at the same locus (all but type I, known to be encoded by a different allele not tested here).

They concluded that the mating locus in the germline nucleus contained six gene pairs, one for each mating type, each comprising an *MTA*-like and *MTB*-like gene, and arranged in the following order: II–V–VI–IV–VII–III. Like *MTA* and *MTB* in the somatic nucleus, these genes were arranged head to head and contain distal transmembrane exons, but with a difference: most of these exons were incomplete, with only the outer two genes (i.e., the *MTA* gene of II and the *MTB* gene of III) having complete exons.

How does *Tetrahymena* create a single *MTA/MTB* pair, of a single mating type, in the somatic nucleus? As the somatic nucleus develops, the authors found, the mating type locus undergoes a series of random cleavage and joining events, ultimately leaving only one pair of genes corresponding to a single mating type at the locus, and deleting the others. But while the central mating type–specific portion of the gene pair remains unchanged by the rearrangements, the authors were able to show that the (incomplete) transmembrane domain-coding segments need to be completed by the generation of chimeric exons using segments from the complete outer genes (*MTA* type II and *MTB* type III).

There is still much to be discovered about this delightfully baroque system. Although exactly what the mating type proteins do is unknown, the discovery of their genetic basis will likely lead quickly to better understanding of their function. And determining how *Tetrahymena* retains the integrity of the mating system genes while subjecting them to such extensive rearrangements is likely to lead to better understanding of DNA editing and repair mechanisms.

**Cervantes MD, Hamilton EP, Xiong J, Lawson MJ, Yuan D, et al. (2013) Selecting One of Several Mating Types through Gene Segment Joining and Deletion in** ***Tetrahymena thermophila*** **. doi:10.1371/journal.pbio.1001518**

